# Aging Is Associated With Multidirectional Changes in Social Cognition: Findings From an Adult Life-Span Sample Ranging From 18 to 101 Years

**DOI:** 10.1093/geronb/gbac110

**Published:** 2022-08-19

**Authors:** Sarah A Grainger, John D Crawford, Julia C Riches, Nicole A Kochan, Russell J Chander, Karen A Mather, Perminder S Sachdev, Julie D Henry

**Affiliations:** School of Psychology, The University of Queensland, Queensland, Australia; Centre for Healthy Brain Ageing (CHeBA), School of Psychiatry, The University of New South Wales, Sydney, Australia; The University of Sydney, Sydney, Australia; Centre for Healthy Brain Ageing (CHeBA), School of Psychiatry, The University of New South Wales, Sydney, Australia; Centre for Healthy Brain Ageing (CHeBA), School of Psychiatry, The University of New South Wales, Sydney, Australia; Centre for Healthy Brain Ageing (CHeBA), School of Psychiatry, The University of New South Wales, Sydney, Australia; Neuroscience Research Australia (NeuRA), Sydney, Australia; Centre for Healthy Brain Ageing (CHeBA), School of Psychiatry, The University of New South Wales, Sydney, Australia; Neuropsychiatric Institute, Princes of Wales Hospital, Sydney, Australia; School of Psychology, The University of Queensland, Queensland, Australia

**Keywords:** Affective empathy, Life-span aging, Social behavior, Social perception, Theory of mind

## Abstract

**Objectives:**

Normal adult aging is associated with changes in social cognition. Although 4 social cognitive domains have been identified (social perception, theory of mind [ToM], affective empathy, and social behavior), no study has tested all 4 domains concurrently in a life-span sample, limiting understanding of the relative magnitude of age-related changes *across* domains. This study addresses this gap by providing the first assessment of all 4 social cognitive domains in an adult life-span sample.

**Methods:**

Three hundred and seventy-two participants ranging from 18 to 101 years of age took part in this study. Participants completed a testing battery that assessed social perception, ToM, affective empathy, and social behavior, as well as broader cognitive function and well-being.

**Results:**

The results showed that adult aging is associated with multidirectional changes in social cognitive abilities, with ToM and social perception showing nonlinear decline across much of the life-span, and affective empathy and social behavior showing improvement. Age remained a significant predictor of all 4 social cognitive domains, even after accounting for broader cognitive function. Weak associations emerged between some of the social cognitive abilities and and indices of broader well-being.

**Discussion:**

These findings provide novel and important evidence that normative aging is associated with both gains and losses in social cognition that occur at distinct points of the adult life-span, and that are at least partially independent of general age-related cognitive decline.

Social cognition—the abilities by which we perceive, interpret, and process social information about ourselves and others—is a fundamental cognitive capacity. In the absence of effective social cognitive skills, developing and maintaining strong social relationships is likely to be challenging, with important implications for broader health and well-being (Holt-Lunstad et al., 2015). There are four core domains of social cognition: social perception, theory of mind (ToM), affective empathy, and social behavior (Henry et al., 2016). The ability to perceive basic social and emotional cues, such as identifying emotional expressions from faces or voices, is referred to as social perception. ToM refers to our capacity to understand others’ mental states and to appreciate that these may differ from our own, and affective empathy refers to one’s emotional response to the perceived situation of another. The way in which one communicates and behaves in social interactions is known as social behavior, with abnormalities manifesting as poor manners and a lack of social tact.

## How Does Normal Aging Relate to Social Cognition?

A central tenet of life-span motivation models is that aging is associated with changes in the prioritization of socioemotional goals, such that as we age, we become less focused on establishing new social contacts and place greater priority on nurturing our close social relationships (Carstensen et al., 1999). Consistent with this view, prior work has shown that we selectively cull our social networks as we age, with older adults reporting smaller but emotionally closer social circles (Carstensen, 1992). Given that meaningful social relationships are prioritized in older age, it might be expected that the core social cognitive capacities that underpin effective social interaction become more fine-tuned, and consequently improve with normal aging. However, aging is associated with changes in the brain regions (e.g., frontal and temporal lobes) that support social cognitive processing (Bartzokis et al., 2001). Thus, from a theoretical perspective, there are two equally important competing forces—motivation and capacity—that have the potential to affect social cognitive abilities as we age, but in different directions (see Henry et al., in press).

There is now a considerable literature showing that normal adult aging is associated with changes in social cognition, with the predominant pattern being one of decline. Importantly though, declines in social cognition do not appear to be equivalent across domains, suggesting that some social cognitive domains may be more sensitive to aging than others. Indeed, while there are well-established declines in social perception (Grainger et al., 2017; Hayes et al., 2020; McKay et al., in press) and ToM (Grainger et al., 2019; Henry et al., 2013), there is less evidence for age-related decline in affective empathy. In fact, affective empathy appears to be the only domain that may show age-related stasis (Bailey et al., 2008, 2020; Beadle & de la Vega, 2019) or potentially even enhancement (Grühn et al., 2008; Sze et al., 2012; Ze et al., 2014). With respect to social behavior, the empirical evidence is more limited, likely due to difficulties associated with its assessment. However, two early studies found that older adults displayed more socially inappropriate behavior than their younger counterparts (Henry et al., 2009; von Hippel & Dunlop, 2005).

Current understanding of the relationship of age with social cognition is limited by a reliance on extreme age-group designs that exclusively compare younger and older adults. While such studies have established how social cognition differs between the early and late stages of the life-span, they provide no insight into *when* age-related social cognitive changes emerge and how they might progress. A small handful of studies have assessed social cognitive function in adult life-span samples but the findings are mixed. Indeed, some studies have shown that social perception and ToM decline from middle age (Bernstein et al., 2011; Mill et al., 2009; Pardini & Nichelli, 2009; Richoz et al., 2018) but others have shown that decline only occurs from old age (Duval et al., 2011; Horning et al., 2012; Phillips et al., 2011; West et al., 2012). The findings are also mixed with respect to affective empathy, with one study showing relative stability (Kelly et al., 2022) and another reporting improvement across the life-span (Sze et al., 2012). Surprisingly, social behavior is yet to be objectively assessed in an adult life-span sample. Taken together, these findings suggest that aging may differentially affect the four social cognitive domains, with some domains showing greater age-related sensitivity and earlier decline, than others.

In prior studies that have used life-span approaches to assess social cognition, typically only a single social cognitive domain has been assessed. Therefore, it is not possible to draw any conclusions regarding the relative developmental differences across the four core domains because sample characteristics have not been held constant. Consequently, the first aim of this study was to investigate the degree to which normal aging affects all four social cognitive domains in an adult life-span sample.

## Does Broader Cognitive Function Account for Any Age-Related Variance in Social Cognitive Function?

Normal aging is associated with declines in cognitive function, such as processing speed, executive functioning, and memory (Ferguson et al., 2021; Salthouse, 2009). While the role of broader cognitive function in social cognitive capacity has been examined previously, the findings have been quite mixed. Some studies have shown that performance on certain cognitive domains accounts for at least some of the age variance in social cognition (Charlton et al., 2009; Kong et al., 2022; Phillips et al., 2011; Rakoczy et al., 2012), but others have shown that age-related social cognitive difficulties can also occur quite independently of broader cognitive losses (Bernstein et al., 2011; Cavallini et al., 2013). Given these mixed findings, our second aim was to examine the extent to which broader cognitive function accounts for age-related variance in each of the social cognitive domains.

## How Is Social Cognition Related to Broader Indicators of Well-Being?

Prior work has shown that social cognitive difficulties are linked to poorer well-being (Grühn et al., 2008; Hall et al., 2009). In older age in particular, ToM performance has been linked to the size of close interpersonal networks (Radecki et al., 2019), friendships (Lecce et al., 2017), and psychological well-being (Lee et al., 2020). While disruptions in any of the four social cognitive domains may negatively affect well-being, at present it is not known which domain might be most important here. The final aim of this study was therefore to provide the first test of how each of the four social cognitive domains is related to measures of well-being.

## Hypotheses

The following hypotheses were preregistered on the Open Science Framework prior to data analysis (https://osf.io/6v2qb/?view_only=123f2e7a981d47f7aac2c34f31d5035d). Overall, we expected age to be negatively associated with ToM, social perception, and social behavior, with the largest age effects expected for the first two domains. Most prior work has shown that affective empathy remains relatively stable throughout the life span; therefore, we expected age to be unrelated to affective empathy. Because the current literature is both limited and mixed with respect to *when* age-related social cognitive changes occur during the adult life span, we did not make any predictions as to whether linear or nonlinear age effects would emerge. Additionally, given the mixed findings with respect to the contribution of broader cognition to age-related social cognitive change, we made no predictions regarding the role of broader cognitive function on social cognition. With respect to associations with well-being, we predicted that social perception and ToM would be associated with lower negative affect (i.e., depression, anxiety) and greater satisfaction with life and social engagement.

## Method

### Participants

In total, 372 participants took part in this study, ranging from 18 to 101 years of age. Demographic characteristics are presented in Table 1. Although age was treated as a continuous variable in this study, the demographic characteristics are presented separately by age group (i.e., young, middle-aged, old, very old) for illustrative purposes. The younger and middle-aged participants (<60 years) were recruited from the local community via advertisements on social media and in local newspapers. All older adults (60 years +) were recruited from ongoing longitudinal studies (see Sachdev et al., 2010, 2013), and via advertisements in the local community. Participants were excluded if they had a current neurological or psychiatric illness or could not speak English fluently. All participants aged 60 years and older were screened for abnormal cognitive decline using the Mini-Mental State Examination (MMSE) and all scored above the recommended age cutoffs.

**Table 1.** Participant Demographic Characteristics Presented for the Complete Sample and Separately by Age Group

Age group (age range)	Full sample (18–101)	Young (18–34)	Middle-aged (35–57)	Old (60–79)	Very old (80–101)
*N*	372	124	114	73	61
Age, mean (*SD*)	49.20 (24.86)	21.69 (3.97)	45.22 (6.12)	69.38 (6.10)	88.39 (4.80)
Female, *n* (%)	241 (65%)	79 (64%)	72 (63%)	55 (75%)	35 (57%)
Education, mean (*SD*)	14.81 (3.21)	14.73 (2.06)	16.07 (3.49)	14.45 (3.56)	13.07 (3.26)
Ethnicity					
Caucasian	80%	65%	85%	82%	98%
Asian	9%	18%	7%	4%	—
Indigenous Australian	1%	—	3%	—	—
Pacific Islander	<1%	2%	—	—	—
Other	10%	15%	5%	14%	2%
Marital status					
Single	46%	76%	47%	15%	20%
Married/de facto	38%	19%	49%	55%	33%
Widowed	8%	—	1%	11%	34%
Other	8%	5%	3%	19%	13%
Health, mean (*SD*)	3.50 (0.87)	3.40 (0.89)	3.40 (0.85)	3.75 (0.81)^a^	3.40 (0.87)^b^

*Notes*: *SD* = standard deviation. “Health” refers to self-rated health, which was measured with a single question “In general, how would you rate your overall health?” and responses were made on a 5-point scale with higher scores indicating better overall health.

^a^ *n* = 71.

^b^ *n* = 51.

### Materials

This study was part of a larger research program and some additional assessments were completed but are not reported here. We preregistered our study design and analytic plan prior to conducting analyses, and this can be accessed via the Open Science Framework (https://osf.io/sedw9/?view_only=123f2e7a981d47f7aac2c34f31d5035d).

#### Social cognition assessments

##### Social perception.

The Emotion Evaluation Test (EET) from The Awareness of Social Inference Test-Short (TASIT-S; Honan et al., 2016) was used to index social perception. This task includes videos of people in everyday scenarios and examines the ability to interpret emotional expressions from paralinguistic and nonverbal cues. The TASIT-S stimuli include male and female actors of Caucasian and Asian backgrounds that vary in age from young to middle adulthood. The EET includes 10 trials and participants are required to select the primary emotion being displayed from a list of seven options (happy, surprised, sad, anxious, angry, revolted, and neutral). Total scores were converted into a percentage accuracy score, with higher scores indicating greater accuracy. TASIT-S has been previously validated in a healthy life-span sample (McDonald et al., 2018).

##### Theory of mind

Part 3 from TASIT-S (Honan et al., 2016) was used to index ToM. This task includes similar videos to the EET but instead measures the ability to make inferences about others’ intentions and beliefs. Specifically, participants are required to differentiate between sarcasm and white lies during conversational exchanges. The task includes nine trials in total and participants are required to respond to four probe questions by answering yes, no, or don’t know. Total scores were converted into a percentage accuracy score with higher scores indicating greater ToM.

##### Affective empathy

The affective empathy component of the Multifaceted Empathy Test (MET; Foell et al., 2018) was used to index affective empathy. This task includes 40 emotionally evocative images of people in a range of contexts that vary in age, gender, ethnicity, and socioeconomic status. Participants are asked to view the images and rate the degree to which they empathized with the protagonist on a scale of 1 (*not at all*) to 9 (*very much*). Prior to completing the task, participants were told that “empathize” refers to feeling or sharing the emotions of the main protagonist. Participants’ ratings across the trials were averaged to create an overall affective empathy score. The MET has been previously used with younger and older adult populations (Ze et al., 2014).

##### Social behavior

The informant-rated Socioemotional Dysfunction Scale (SDS; Barsuglia et al., 2014) was used to index social behavior. The SDS includes 40 statements that are designed to measure a range of behaviors including: extraversion, warmth, social influence, insight, openness, appropriateness, and maladjustment. Statements include, “The participant often makes people uncomfortable” and “The participant often makes social errors.” Informants are required to rate each statement on a 5-point scale, with 1 indicating the statement is very inaccurate and 5 indicating that it is very accurate. The SDS provides a global score of social competencies, with higher scores indicative of poorer social behavior. However, we reverse-coded scores on the SDS so that higher scores indicated better social behavior.

#### Cognitive assessments

We used a comprehensive neuropsychological test battery that indexed five cognitive domains: processing speed, attention, working memory, episodic memory, executive function. Raw scores for each of the individual cognitive assessments were transformed into *z*-scores. Where necessary, the signs of the *z*-scores were reversed so that higher scores reflected better performance. Composite scores were created to index broader cognitive domains, by creating a mean of the *z*-scores, and then transforming that score into a *z*-score. Composite variables were only created if the individual measures were correlated strongly with each other (i.e., *r* ≥ 0.50). Means and standard deviations for each of the cognitive domains are presented in Table 2 and correlations between each of the cognitive domains are presented in Supplementary Table 1.

**Table 2.** Descriptive Statistics for the Social Cognitive and Cognitive Domains, Presented Separately by Age Group

Age group (age range)		Young (18–34)	Middle-aged (35–57)	Old (60–79)	Very old (80–101)
	*N*	Mean (*SD*)	Mean (*SD*)	Mean (*SD*)	Mean (*SD*)
Social cognition					
Social perception	372	83.51 (11.04)	78.60 (12.32)	70.79 (17.04)	49.51 (18.83)
ToM	371	83.56 (9.20)	82.85 (9.11)	74.85 (11.79)	63.68 (11.05)
Affective empathy	362	5.74 (1.32)	6.33 (1.32)	6.54 (1.42)	6.63 (1.24)
Social behavior^a^	191	164.92 (22.28)	169.89 (24.22)	176.79 (21.30)	174.50 (23.74)
Cognition^b^					
Processing speed	340	0.72 (0.54)	0.25 (0.55)	−0.48 (0.68)	−1.60 (0.73)
Attention	340	0.20 (0.93)	−0.04 (1.00)	−0.11 (1.08)	−0.21 (1.03)
Working memory	340	0.21 (1.03)	0.00 (1.02)	−0.11 (0.85)	−0.15 (0.97)
Episodic memory	340	0.28 (0.94)	0.29 (0.80)	−0.11 (0.79)	−1.05 (0.97)
Executive function	340	0.61 (0.60)	0.30 (0.62)	−0.25 (0.63)	−1.52 (0.97)

*Notes*: *SD* = standard deviation; ToM = theory of mind.

^a^Some participants did not return their informant-report assessments, which resulted in a reduced sample size for social behavior.

^b^Means for the cognitive domains are expressed as *z*-scores. Age is used as a continuous variable in all analyses but scores are presented separately by age group for illustrative purposes.

The WAIS-III Coding and Trail Making Test (Part A) were used to index processing speed. Attention was indexed via the WAIS-III Digit Span Forward, and working memory was indexed using the Wechsler Adult Intelligence Scale Third Edition (WAIS-lll) Digit Span Backward. Episodic memory was indexed using the Logical Memory Story A immediate and delayed recall. Finally, executive function was indexed using semantic fluency (Animals), Trail Making Test (Part B), and Stroop Interference (see Author Note 1).

#### Well-being measures

##### Psychological well-being

The Hospital Anxiety and Depression Scale (HADS, Zigmond & Snaith, 1983) was used to index symptoms of depression and anxiety. It includes 14 statements in total, and responses are made on a 4-point scale. We calculated total scores separately for anxiety and depression, with higher scores indicating greater negative affect.

##### Social well-being

The Lubben Social Network Scale (LSNS; Lubben, 1988) was used to index social engagement. The LSNS includes 12 statements total, with six of the statements enquiring about engagement with family and the remaining six enquiring about engagement with friends. We calculated separate total scores for engagement with family and friends. Responses are made on a 6-point scale and higher scores indicate greater social engagement.

##### General well-being

The Satisfaction with Life Scale (Diener et al., 1985) was used to index general subjective well-being. It includes five statements that enquire about one’s life satisfaction and responses are made on a 7-point scale. Higher scores indicate greater well-being.

### Procedure

All participants were asked to provide written informed consent on arrival. Older adults recruited from the community completed the MMSE to screen for abnormal cognitive decline. Older adults recruited from the longitudinal aging studies had recently completed the MMSE and scored above the recommended cutoff for cognitive impairment. Next, all participants were randomly assigned to complete one of four counterbalanced orders of the tasks. All demographic questions, social cognitive tasks (except the informant-rated measure of social behavior), and social function measures were presented on a laptop computer via the Qualtrics platform, and all cognitive assessments were completed via pen and paper tests that were administered by trained research assistants. Informant-rated assessments were completed using pen and paper, and were given to participants at the end of the testing session in an envelope along with instructions on how these should be completed. Specifically, participants were instructed to ask someone who knows them well (e.g., a close friend or family member) to complete the questionnaires independently on their behalf. The testing session took approximately 3 hrs to complete for younger and middle-aged adults, and slightly longer for the older adult cohort, but this was inclusive of frequent breaks. All participants were compensated $60 for their time. This study was approved by The University of Queensland Human Research Ethics Committee (Approval number: 2017000770).

### Analytic Approach

The first set of analyses involved testing whether age was a predictor of social cognition, and whether there were any statistically significant nonlinear effects. To achieve this, we conducted four separate multiple regression analyses (i.e., one for each of the four social cognitive domains as dependent variables) with age (mean-centered) and age (mean-centered)-squared as predictors. During the peer-review process, it was suggested that key sociodemographic variables should be controlled for in these analyses; therefore, we also ran these analyses while controlling for sex and education. These results are presented in Supplementary Table 2. Importantly, none of the conclusions changed when these control variables were included in the analyses.

The next analyses aimed to assess the contribution of age when broader cognition was accounted for in the model. A small proportion of the sample did not have cognition data available (*n* = 32), so we first conducted a regression analysis (for each social cognitive domain) with age and age-squared using only the participants that had complete social cognition and cognition data available (Model 1). This allowed us to assess the proportion of variance accounted for by age in this slightly reduced sample size. Following this, we conducted a hierarchical multiple regression analysis with the cognitive domains entered in the first step (Model 2) followed by age and age-squared in Step 2 (Model 3). To evaluate the relative contribution of broader cognitive function, we compared the variance explained by age in Models 1 and 3.

To assess the relationships between social cognition and well-being, we conducted partial correlations between each social cognitive domain and the five well-being indicators controlling for age. The decision to control for age in these correlational analyses was made post hoc, based on prior work that has shown that age is differentially associated with both social cognition and well-being.

## Results

Descriptive statistics for each of the four social cognitive domains are presented in Table 2.

### Social Perception

**Figure 1.**
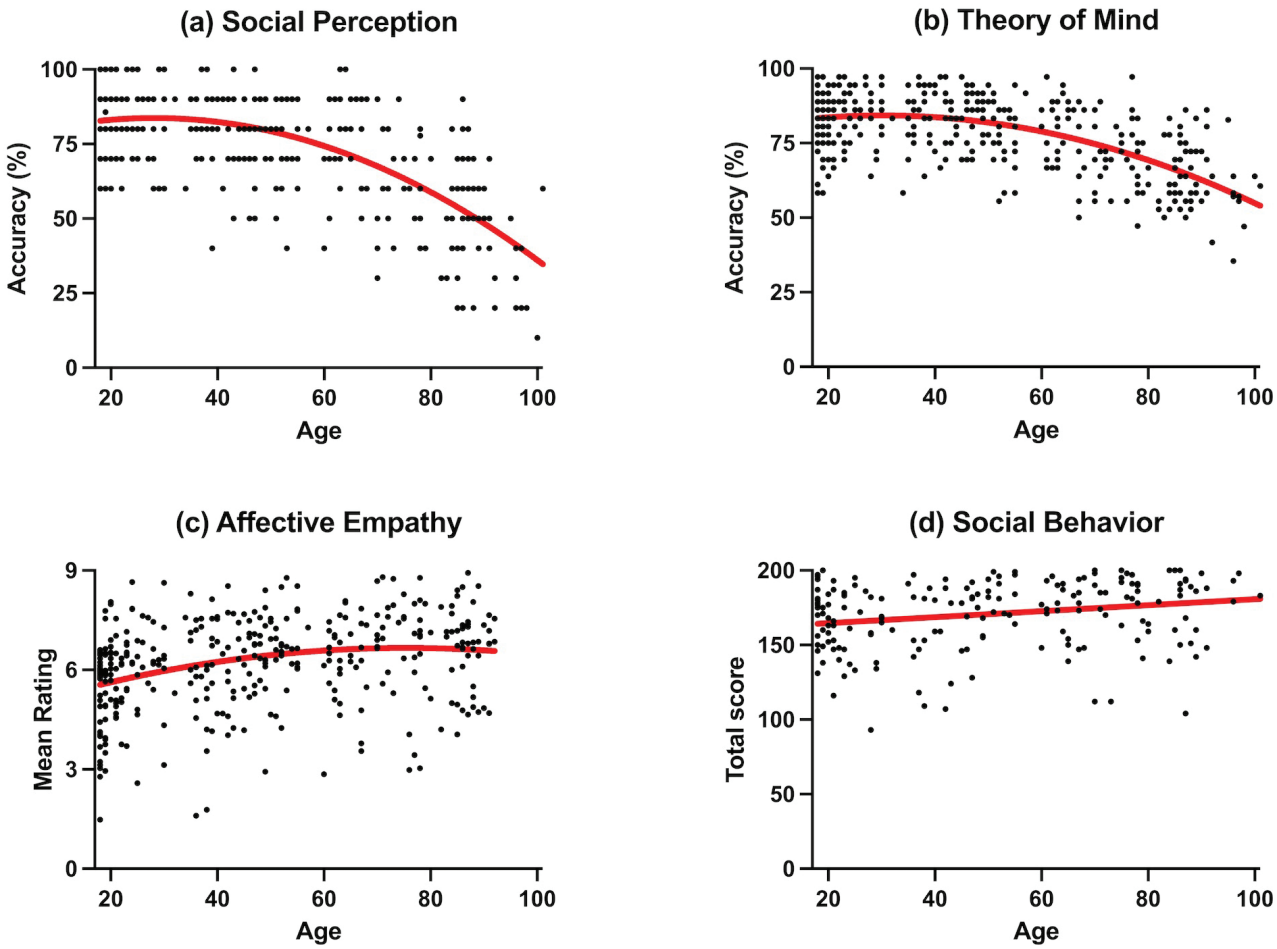
The association between age and (A) social perception, (B) theory of mind, (C) affective empathy, (D) social behavior.

As can be seen in Table 3, both the linear and quadratic effects of age were significant independent predictors of social perception, with the model explaining 46% of the variance in social perception scores. Figure 1A shows that social perceptual abilities appear to remain relatively stable in younger adulthood and early middle adulthood but then show an accelerating rate of decline from around 40 years of age.

**Table 3.** Regression Analyses for Linear and Quadratic Effects of Age on Each of the Social Cognitive Domains

	Social perception		Theory of mind		Affective empathy		Social behavior	
	*B*	*t* (*p*)	*B*	*t* (*p*)	*B*	*t* (*p*)	*B*	*t* (*p*)
Age(cnt)	−0.39	12.86 (<.001)	−0.23	10.47 (<.001)	0.02	5.94 (<.001)	0.20	2.85 (.005)
Age(cnt)^2^	−0.01	6.82 (<.001)	−0.01	6.18 (<.001)	0.00	2.31 (.022)	0.00	0.02 (.986)
*R* ^2^, *F* (*p*)	0.46, 156.33 (<.001)		0.37, 109.98 (<.001)		0.09, 18.10 (<.001)		0.05, 4.50 (.012)	

*Note*: Age(cnt): age centered at its mean value of 49.20.

Next, we examined the contribution of broader cognitive function to the relationship between age and social perception. Model 2 revealed that the cognitive domains were significantly associated with social perception scores, accounting for 31% of the variance. Processing speed, episodic memory, and executive function were significant independent predictors of social perception scores. Introducing age and age-squared at Step 2 (Model 3) explained an additional 5% of the variance. These findings indicate that the inclusion of broader cognitive function in the model reduced the variance explained by age (i.e., age and age-squared) from 34% to 5% (see Supplementary Table 3). While age and age-squared were significant independent predictors of social perception scores, none of the cognitive domains remained significant independent predictors when age was included in the model.

### Theory of Mind

For ToM, the linear and quadratic effects of age were significant independent predictors, with the model explaining 37% of the variance in ToM scores (see Table 3). Figure 1B shows that ToM appears to remain relatively stable in younger and middle adulthood and then begins to decline from around 50 years of age.

Next, we examined the contribution of broader cognitive function to the relationship between age and ToM (see Supplementary Table 4). Together, the cognitive domains were significantly associated with ToM scores (Model 2), accounting for 27% of the variance. Processing speed, episodic memory, working memory, and executive function were all unique predictors of ToM performance. Introducing age and age-squared (Model 3) explained an additional 6% of the variance. These findings indicate that when broader cognitive function is taken into account, the variance explained by age (i.e., age and age-squared) is reduced from 30% to 6%. Age and age-squared were significant independent predictors of ToM, but only attention and executive function were unique predictors of ToM when the age variables were included in the model.

### Affective Empathy

The linear and quadratic effects of age were both significant independent predictors of affective empathy, with the model explaining 9% of the total variance in affective empathy scores (see Table 3).

As can be seen in Figure 1C, affective empathy appears to increase throughout young, middle, and early older adulthood and then plateaus in very old age. As can be seen in Supplementary Table 5, broader cognitive function was significantly associated with affective empathy, explaining 6% of the variance in affective empathy scores. However, only processing speed was a significant independent predictor of affective empathy. Introducing age and age-squared (Model 3) accounted for a further 4% of the variance in affective empathy scores. These findings show that when broader cognitive function is accounted for, the variance explained by age (i.e., age and age-squared) is reduced from 9% to 4%. Age (but not age-squared) was the only significant predictor of affective empathy when the age and cognitive variables were included in the model.

### Social Behavior

As can be seen in Table 3, age was significantly associated with social behavior, explaining approximately 5% of the variance. However, only the linear effect of age was a significant independent predictor of social behavior. Figure 1D shows that social behavior improves across the life span in a linear fashion.

As can be seen in Supplementary Table 6, the cognitive variables entered together were significantly associated with social behavior, accounting for 8% of the variance on social behavior scores (Model 2). Processing speed was the only unique predictor of social behavior. The inclusion of age and age-squared (Model 3) explained an additional 3% of the variance, although this was not significant. These results show that when broader cognitive function is considered, age does not explain additional variance in social behavior scores. However, age was the only significant independent predictor of social behavior when all variables were included in the model.

### Correlational Analyses

We conducted partial correlations between social cognition and five measures of well-being, while controlling for age. As can be seen in Table 4, weak correlations were identified between some of the social cognitive domains and well-being measures.

**Table 4.** Partial Correlations Between Each of the Four Social Cognitive Domains and Well-Being Indices, Controlling for Age

Variable	Anxiety	Depression	Social engagement (family)	Social engagement (friends)	Life satisfaction
Social perception	−0.01	−0.13*	0.05	0.15**	0.00
ToM	0.05	−0.02	0.12*	0.07	0.03
Affective empathy	0.11*	−0.03	0.05	−0.05	0.12*
Social behavior	−0.07	−0.02	0.16*	0.17*	0.09

*Notes*: ToM = theory of mind.

*p < .05. **p < .01.

## Discussion

Prior literature suggests that normal adult aging is associated with changes in social cognitive function. While four domains of social cognitive function have been identified, these have typically been assessed individually, making it difficult to compare the relative effects of aging across social cognitive domains. The present study was therefore important because it provided the first investigation of all four social cognitive domains concurrently in the same community representative life-span sample.

The first key finding was that aging is associated with multidirectional changes in social cognition. Whereas aging was negatively associated with ToM and social perception, it was positively associated with affective empathy and social behavior. This finding of age-related decline in both social perception and ToM was in line with our predictions and consistent with broader literature (Grainger et al., 2017; Phillips et al., 2011; Richoz et al., 2018). However, the age-related enhancement of affective empathy was not anticipated, nor was the improvement in social behavior. We expected age to be unrelated to affective empathy based on prior work that has identified age-related stasis in this domain; however, the finding of age-related improvement does align with prior studies that found improved affective empathic capacity in older age (Sze et al., 2012; Ze et al., 2014), as well as with theoretical models of aging that predict enhancement of socioemotional functioning in older age (Carstensen et al., 1999). However, it is important to note that age accounted for only 9% of the total variance in affective empathy scores, which is small relative to the age effects identified for social perception and ToM. Several prior studies in the affective empathy literature have included sample sizes that are underpowered to detect small effects (Bailey et al., 2008, 2020), and this may explain some of the previously identified null age effects. With respect to social behavior, we expected to see declines across the adult life-span but instead we found improvement. Out of the four social cognitive domains, social behavior has been the focus of the least empirical study to date. However, the two prior studies that tested whether there are age differences in social behavior found that older age is associated with an increase in social behavioral abnormalities, suggesting this domain is impaired with normal aging (Henry et al., 2009; von Hippel & Dunlop, 2005). However, both of these studies focused specifically on social inappropriateness to index social behavior, whereas the SDS used in the current study tapped into a broader range of social behavior dimensions that included not only social inappropriateness, but also warmth, extraversion, social influence, insight, openness, and maladjustment (Barsuglia et al., 2014). The current findings therefore speak to the possibility that the way in which social behavior is operationalized may determine not only the magnitude but the direction of age effects.

### The Complex Nature of Age Effects

One strength of this study’s design was the inclusion of a large life-span sample that allowed us to test whether aging exhibited linear or nonlinear effects on social cognitive function. The results show a complex profile of age effects, providing evidence for linearity, nonlinearity, losses, and gains.

While significant nonlinear age effects emerged for ToM, social perception, and affective empathy, the pattern of age-related change differed meaningfully across these domains. Whereas age-related change was evident around middle age for social perception (i.e., 40 years) and declined quite steadily from this point, ToM showed relative stability throughout young and middle adulthood, with changes emerging at around 50 years of age, after which decline occurred more gradually across the life-span. These findings suggest that although social perception and ToM are both susceptible to age-related decline, ToM may be more resilient to the effects of aging until more advanced stages of late-life development. Moreover, affective empathy appears to be more resilient still; performance on this domain increased throughout early, middle and older adulthood until finally plateauing in very late adulthood. These data suggest that affective empathy may be quite unique from most other cognitive abilities, in that it continues to develop throughout most of the life-span. Finally, social behavior was the only social cognitive domain that did not display a nonlinear effect of aging—instead showing an age-related linear increase, suggesting that there may be continual improvements in our ability to regulate our social behavior. Taken together, these findings suggest that when considered overall, social cognitive abilities may be relatively resilient to the effects of aging, particularly in contrast to broader cognitive abilities, where age-related declines are often evident by our 20s and 30s (Ferguson et al., 2021; Salthouse, 2009).

### The Contribution of Broader Cognitive Decline to Social Cognitive Aging

The second aim of this study was to examine whether aging is associated with social cognition after accounting for broader cognitive function. Although cognitive function did account for significant variance in all four social cognitive domains, age accounted for additional unique variance in social perception, ToM, and affective empathy scores. Importantly though, age was consistently the strongest independent predictor of all four social cognitive domains when broader cognition was accounted for. These findings add to a growing literature showing that changes in broader cognitive function contribute to social cognitive aging but are not the sole factor responsible for the observed age effects (Kong et al., 2022; Orgeta & Phillips, 2008; Phillips et al., 2011).

As noted earlier, motivational models of aging argue that older age is associated with a tendency to prioritize meaningful social relationships (Carstensen, 1992). Despite the fact that older adults are intrinsically motivated to nurture their social relationships, the current findings indicate that they exhibit difficulties in some of the underlying skills required to have effective social interactions (i.e., understanding others’ mental and emotional states) and that these difficulties are partially independent of general age-related cognitive decline. However, these models of aging also argue that older adults have less motivation to expend resources on activities that are not meaningful to them (see Henry et al., in press). Therefore, age-related changes in motivation to do well on the task might also have contributed to older adults’ poorer performance on the ToM and social perception tasks. Indeed, prior work has shown that age effects are reduced when social cognitive tasks are designed to be more meaningful to older adults (Stanley & Isaacowitz, 2015). An important next step in this literature will be to move social cognitive assessment outside of the laboratory and into naturalistic settings where older adults’ social cognitive abilities can be measured during genuine social interactions.

### Social Cognition and Well-Being

Finally, contrary to our predictions, there was little evidence to suggest that social cognitive function is linked to well-being. Although a few correlations emerged between some of the social cognitive domains and well-being outcomes, these were very weak in magnitude, failing to provide strong evidence that any particular social cognitive domain is strongly linked to broader well-being. The lack of robust associations between well-being measures is surprising, given that prior work has established links between social cognition and broader well-being (Grühn et al., 2008; Lecce et al., 2017; Lee et al., 2020).

### Limitations

Although this study was strengthened by a large well-powered design, there are limitations need to be acknowledged. Firstly, an informant-report assessment was used to index social behavior and as a result there were some missing data for this social cognitive domain. While missing data are very common with this assessment type, informant-report was considered critical for measuring social behavior because it provides an independent and objective evaluation, which unlike self-report, is not reliant on intact self-awareness and insight. Secondly, the current study design was cross-sectional; therefore, it is not possible to rule out potential cohort effects in responses, especially given the profound social and societal changes that have occurred in the past century. We would therefore encourage future studies focused on social cognitive aging to consider longitudinal designs so that potential cohort effects can be avoided and the trajectory of age-related change across the life span can be better understood. Thirdly, our sample lacked ethnic diversity, particularly in the very old age group (i.e., 80 years +). However, this has been acknowledged as a broad limitation in the current social cognitive aging literature (see Hamilton et al., 2022), and we therefore would encourage future studies to prioritize testing more ethnically diverse older adult samples. Finally, although the four social cognitive domains were differentially affected by aging in the current study, it is important to acknowledge that different forms of assessment were used to index each of the four domains. Whereas social perception and ToM were indexed using objective assessments, affective empathy was indexed via self-report, and social behavior via informant-report. While all of the measures used in the current study are regarded as the most validated for their respective domains (see Henry et al., 2016), it is not possible to rule out variance due to measurement type contributing to the observed age effects. Future studies could address this limitation by using multimethod approaches to measure each of the social cognitive domains.

## Conclusion

By providing the first evaluation of all four social cognitive domains in a single life-span cohort, this study provides important new knowledge about the nature of age-related change in social cognition. It revealed that aging is associated with both losses and gains in social cognition, that these occur at different stages of the adult life span and at different rates, and at are at least partially independent of age-related changes in broader cognitive function.

## Supplementary Material

gbac110_suppl_Supplementary_Material

## Data Availability

Our study design and hypotheses were preregistered on the Open Science Framework prior to conducting any analyses. Our data, analytic methods, and materials are available to other researchers on request. Please contact the corresponding author (S. A. Grainger) to access any material from this study.

## Author Note

1. We planned to include WAIS-III Similarities and phonemic fluency in the executive function composite but they did not correlate strongly with the other measures in the composite and therefore were excluded.
