# Supplementary material for: Aging Is Associated With Multidirectional Changes in Social Cognition: Findings From an Adult Life-Span Sample Ranging From 18 to 101 Years
Source: J Gerontol B Psychol Sci Soc Sci. 2022 Aug 19;78(1):62–72. doi: 10.1093/geronb/gbac110 (PMC9890910; doi:10.1093/geronb/gbac110)
Supplement: gbac110_suppl_Supplementary_Material [file gbac110_suppl_supplementary_material.pdf]

Supplementary Material

eTable 1
Pearson Correlations Between Each of the Five Cognitive Domains

	1	2	3	4	5
Processing speed	1	.19***	.21***	.43***	.77***
Attention		1	.54***	.14*	.32***
Working memory			1	.24***	.23***
Episodic memory				1	.48***
Executive function					1

$p < .05$, $p < .001$

e-Table 2

Regression Analyses with Linear and Quadratic Effects of Age on Each of the Social Cognitive Domains, controlling for Sex and Education

	Social perception		Theory of Mind		Affective Empathy		Social Behavior	
	B	*t* (*p*)	B	*t* (*p*)	B	*t* (*p*)	B	*t* (*p*)
Education	1.04	3.60 (<.001)	.76	3.90 (<.001)	.03	1.29 (.198)	-.05	.09 (.931)
Sex	4.70	2.47 (.014)	2.62	2.05 (.041)	.06	0.42 (.676)	4.33	1.28 (.203)
ΔR^2 F (p)	.05, 9.68 (<.001)		.05, 9.90 (<.001)		.01, 0.93 (.397)		.01, 0.82 (.442)	
Age(cnt)	-.39	12.86 (<.001)	-.23	10.40 (<.001)	.02	6.03 (<.001)	.21	3.00 (.003)
Age(cnt)^2	-.01	6.36 (<.001)	-.01	5.56 (<.001)	.00	1.81 (.072)	.001	0.22 (.823)
ΔR^2 F (p)	.42, 143.77 (<.001)		.33, 97.49 (<.001)		.09, 18.37 (<.001)		.05, 5.07 (.007)	
R^2 F (p)	.47, 80.47 (<.001)		.38, 56.29 (<.001)		.10, 9.69 (<.001)		.06, 2.96 (.021)	

e-Table 3

Regression Analyses Examining the Association Between (a) Age and Social Perception (Model 1), (b) Cognitive Function and Social Perception (Model 2), and (c) Age and Social Perception While Accounting for Cognitive Function (Model 3).

	Model 1			Model 2			Model 3		
Independent Variable	B	*t*	*p*	B	*t*	*p*	B	*t*	*p*
Processing Speed				4.81	3.93	<.001	0.98	0.71	.481
Attention				1.54	1.76	.080	1.65	1.95	.052
Working Memory				-1.50	1.69	.092	-0.86	0.99	.322
Episodic Memory				1.98	2.27	.024	1.41	1.65	.100
Executive Function				4.11	3.12	.002	2.18	1.64	.101
Age(cnt)	-.36	11.45	<.001				-.25	5.00	<.001
Age(cnt)^2	-.01	4.73	<.001				-.01	3.00	.003
R^2 F (p)	.34, 85.06 (<.001)			.31, 30.58 (<.001)			.37, 27.63 (<.001)		
ΔR^2 F (p)							.05, 14.22 (<.001)		

Note. N = 340. Age(cnt): Age centred at its mean value of 49.20.

e-Table 4

Regression Analyses Examining the Association Between (a) Age and ToM (Model 1), (b) Cognitive Function and ToM (Model 2), and (c) Age and ToM While Accounting for Cognitive Function (Model 3).

	Model 1			Model 2			Model 3		
Independent Variable	B	*t*	*p*	B	*t*	*p*	B	*t*	*p*
Processing Speed				2.16	2.37	.018	-0.51	0.50	.620
Attention				1.23	1.89	.060	1.37	2.18	.030
Working Memory				-1.62	2.46	.015	-1.10	1.71	.089
Episodic Memory				1.70	2.62	.009	1.19	1.87	.062
Executive Function				3.59	3.65	<.001	2.05	2.08	.038
Age(cnt)	-.23	9.71	<.001				-0.20	7.58	<.001
Age(cnt)^2	-.01	5.62	<.001				-0.01	4.89	<.001
R^2 F (p)	.30, 71.12 (<.001)			.27, 24.83 (<.001)			.33, 23.52 (<.001)		
ΔR^2 F (p)							.06, 15.03 (<.001)		

Note. N = 340. Age(cnt): Age centred at its mean value of 49.20.

e-Table 5

Regression Analyses Examining the Association Between (a) Age and Affective Empathy (Model 1), (b) Cognitive Function and Affective Empathy (Model 2), and (c) Age and Affective Empathy While Accounting for Cognitive Function (Model 3).

	Model 1			Model 2			Model 3		
Independent Variable	B	*t*	*p*	B	*t*	*p*	B	*t*	*p*
Processing Speed				-.33	2.67	.008	-.12	0.87	.387
Attention				-.17	1.91	.057	-.15	1.70	.089
Working Memory				-.02	0.19	.851	-.02	0.21	.831
Episodic Memory				-.05	0.52	.606	-.06	0.71	.479
Executive Function				.10	0.71	.476	.12	0.86	.392
Age(cnt)	.02	5.48	<.001				.01	2.85	.005
Age(cnt)^2	.00	2.07	.039				.00	1.88	.062
R^2 F (p)	.09, 15.97 (<.001)			.06, 4.37 (.001)			.10, 5.38 (.001)		
ΔR^2 F (p)							.04, 7.50 (.001)		

Note. Age(cnt): Age centred at its mean value of 49.20. N = 340

e-Table 6
Hierarchical Regression Analysis for the Effect of Age on Social Behavior, Controlling for Cognitive Domains.

	Model 1			Model 2			Model 3		
Independent Variable	B	*t*	*p*	B	*t*	*p*	B	*t*	*p*
Processing Speed				-7.20	2.41	.017	-2.49	0.69	.491
Attention				2.18	1.08	.281	2.27	1.13	.261
Working Memory				-1.32	0.63	.533	-1.54	0.74	.463
Episodic Memory				2.32	1.15	.252	2.99	1.47	.144
Executive Function				-.02	0.01	.995	1.35	0.38	.702
Age(cnt)	.26	3.70	<.001				.28	2.30	.023
Age(cnt)^2	.00	0.74	.463				.00	0.90	.371
R^2 F (p)	.08, 7.51 (.001)			.08, 2.75 (.020)			.11, 2.77 (.009)		
ΔR^2 F (p)							.03, 2.69 (.071)		

Note. N = 174. Age(cnt): Age centred at its mean value of 49.20.
